# Visual cues for manual control of headway

**DOI:** 10.3389/fnbeh.2013.00045

**Published:** 2013-05-21

**Authors:** Simon G. Hosking, Catherine E. Davey, Mary K. Kaiser

**Affiliations:** ^1^Air Operations Division, Defence Science and Technology OrganisationFishermans Bend, VIC Australia; ^2^Human Systems Integration Division, NASA Ames Research CenterMoffett Field, CA, USA

**Keywords:** visual perception, manual control, headway regulation, optic cues, control dynamics

## Abstract

The ability to maintain appropriate gaps to objects in one's environment is important when navigating through a three-dimensional world. Previous research has shown that the visual angle subtended by a lead/approaching object and its rate of change are important variables for timing interceptions, collision avoidance, continuous regulation of braking, and manual control of headway. However, investigations of headway maintenance have required participants to maintain a fixed distance headway and have not investigated how information about own-speed is taken into account. In the following experiment, we asked participants to use a joystick to follow computer-simulated lead objects. The results showed that ground texture, following speed, and the size of the lead object had significant effects on both mean following distances and following distance variance. Furthermore, models of the participants' joystick responses provided better fits when it was assumed that the desired visual extent of the lead object would vary over time. Taken together, the results indicate that while information about own-speed is used by controllers to set the desired headway to a lead object, the continuous regulation of headway is influenced primarily by the visual angle of the lead object and its rate of change. The reliance on visual angle, its rate of change, and/or own-speed information also varied depending on the control dynamics of the system. Such findings are consistent with an optimal control criterion that reflects a differential weighting on different sources of information depending on the plant dynamics. As in other judgements of motion in depth, the information used for controlling headway to other objects in the environment varies depending on the constraints of the task and different strategies of control.

## 1. Introduction

The ability to maintain appropriate gaps to objects in one's environment is important when navigating through a three dimensional world. Indeed, when performing high speed tasks, such as driving vehicles or piloting aircraft, successful gap maintenance and collision avoidance is critical. We aimed to determine the visual cues that people use to regulate gaps between themselves and other objects. In particular, we were interested in the visual cues that are used by operators to control and maintain headway. The experiment that follows investigated two candidate visual cues that may provide information for the control of headway, namely: (1) the retinal image size of the lead object (and its rate of change); and (2) the global optic flow rate (GOFR) available from the projection of ground texture on the retina as one traverses in three-dimensional space.

Previous studies of the visual cues used for the regulation of headway have primarily been carried out in the context of driving and pedestrian behavior. Of these, car following behavior (i.e., the adjustment of driving speed in response to an accelerating/decelerating lead vehicle) has been the focus of extensive research and modeling [for a recent review see Andersen and Sauer (2007)]. Many of the traditional car-following control inputs used to model traffic flow have assumed that headway maintenance is based on physical variables such as relative distance, relative velocity, or relative acceleration [see (Brackstone and McDonald, 1999)]. However, when modeling human controllers, it is important to take into account that information about distance and speed is derived from optical variables (e.g., Fajen, 2005). On this basis, it has been proposed that the visual information used for the regulation of headway is primarily based on the visual angle (θ) subtended by the lead object and/or its rate of change ($\dot{\theta }$).

Lee (1976) proposed that visual information for car following and braking to avoid collision could be derived from the ratio of θ to $\dot{\theta }$ (i.e., what Lee termed *tau*, τ) and its time derivative ($\dot{\tau }$). He demonstrated that, in principle, different types of collision and recession courses that occur when following a vehicle (and hence the required deceleration or acceleration that the driver should perform) are specified by different criterion values of τ and $\dot{\tau }$. It has been suggested that $\dot{\tau }$ is used as information for the initiation and continuous control of braking (Yilmaz and Warren, 1995), and as a variable for maintaining constant time-headway based on time-to-collision (van Winsum and Heino, 1996). However, while a large body of research has been devoted to test Lee's hypothesis that τ is used for interceptions [see (Hecht and Salvelsbergh, 2004)] and collision avoidance (e.g., Yilmaz and Warren, 1995), to our knowledge there has not been any research on whether drivers actually use τ and $\dot{\tau }$ as visual cues for controlling headway.

Lee's (1976) model of car following is problematic for a number of reasons (e.g., see Kaiser and Phatak, 1993; Wann, 1996; Treslian, 1999; Kaiser and Johnson, 2004). Firstly, there are instances during following events where τ and/or $\dot{\tau }$ either can not be calculated or are required to be held at infinity (e.g., when $\dot{\theta }=0$ or when $\ddot{\theta }=0$). Secondly, it is questionable whether participants are sensitive to optic acceleration as is assumed in the $\dot{\tau }$ approach (e.g., see Calderone et al., 1989; Werkhoven et al., 1992; Dubrowski and Carnahan, 2002). Thirdly, the assumptions constraining the τ approach restrict its utility in natural environments that do not conform to such constraints (e.g., Treslian, 1999). Finally, there is mounting evidence that judgements of motion in depth are based on multiple sources of information depending on the constraints of the task and/or the control strategies being used (e.g., DeLucia et al., 2003; Flach et al., 2011).

An alternative model has assumed that regulation of headway is based solely on the $\dot{\theta }$ of the lead object. According to this approach, actors attempt to match their speed to that of a lead object by maintaining $\dot{\theta }=0$ (e.g., Lee and Jones, 1967; van Winsum, 1999). For example, Rio and Warren (2011) found that when pedestrians were asked to walk behind an moving object in a virtual environment, they changed their walking speed on the basis of $\dot{\theta }$, and were not influenced by manipulations of the binocular disparity of the lead object. Lee and Jones (1967) found that the accelerations and decelerations of drivers in naturalistic traffic conditions could be modeled on the same control law. However, Andersen and Sauer (2007) have noted that matching speed to a lead object is not sufficient for car following, particularly when one is following closely behind a lead vehicle that brakes suddenly. They extended the $\dot{\theta }$ model of car following to also take into account the distance of the lead object on the basis of the θ it subtends (θ is inversely proportional to the distance of a lead object). It was proposed that drivers' change in speed (i.e., acceleration, $\ddot{x}$) is the result of the weighted combination of the difference between the θ of the lead object and a desired θ (θ′; which reflects the ideal following distance that a driver attempts to maintain), and the $\dot{\theta }$ of the lead object, such that

(1) $$\ddot{x}=j\left(\frac{1}{\theta }-\frac{1}{\theta^{\prime }}\right)+k\dot{\theta }.$$

Andersen and Sauer (2007) validated their car-following model by measuring drivers' following behavior in both a driving-simulator experiment and in an instrumented-vehicle study. During the simulation trials, the speed of a lead vehicle was continuously varied, and drivers were required to follow at a constant distance. The results of their study found that the optic-based model described in Equation 1 was a better predictor of car-following behavior than an alternative model based on the physical variables of distance and/or speed. The data from naturalistic driving conditions confirmed these results. Other attempts to model operators' control functions when following a lead vehicle with sinusoidal changes (disturbances) to velocity have found that greater following distances led to less control effort and more variation in time headway (Mulder et al., 2005). Mulder et al. (2005) concluded that this was due to a decreased ability to perceive changes in the speed of the lead vehicle at greater viewing distances, presumably because $\dot{\theta }$ was less salient.

Given that participants in the car-following studies described above were required to maintain a constant following distance (and hence a constant θ), the question remains whether following behavior without such constraints could be modeled similarly. Claims that drivers might base their headway on attempts to maintain a constant time headway rather than distance (e.g., Lee, 1976), suggests that a desired θ would vary as a function of following speed (Andersen and Sauer, 2007). Andersen and Sauer (2007) addressed this by proposing that the constant θ′ in Equation 1 could be redefined as one that varies as a function of the size (*s*) of the lead vehicle, the desired time headway (*T*), and the speed of the follower (*V*):

(2) $$\theta^{\prime }=2\cdot \text{ atan}\left(\frac{s}{TV}\right)$$

However, to our knowledge, this extension of Andersen and Sauer's model has not been empirically tested.

How might one determine an appropriate time headway? Gibson and Crooks (1938) proposed that both the perceived speed of the follower and the dynamical properties of the system (e.g., braking ability) are taken into account when performing a following task. Indeed, research on manual control has shown that control dynamics are a strong determinant of human performance (e.g., McRuer and Jex, 1967). For example, Stanard et al. (1996) used a collision-avoidance task to show that human performance will adapt to the demands associated with changing dynamics. When participants in their study were required to use an acceleration-control system, collision-avoidance actions were initiated at a constant time-to-contact. However, when the control response was proportional to position, participants initiated avoidance actions at a constant distance-to-contact. It seems plausible that the information used to control headway would be similarly influenced by response dynamics, in particular as to how these dynamics affect the acceleration capability of the system.

Information about own-speed is, in principle, available in the optic array from both edge rate and GOFR (e.g., Denton, 1980; Larish and Flach, 1990; Andersen et al., 1999; Fajen, 2005; Rock and Harris, 2006; Flach et al., 2011). Edge rate is defined as the number of edges or discontinuities that pass a reference point per unit time (Andersen et al., 1999). For most driving situations, ground surface texture dominates the optical field. GOFR is the rate of optical motion of texture elements in a given visual direction, and it is proportional to ground speed and inversely proportional to eye-height, such that:

(3) $$\text{GOFR}=\frac{V}{h},$$

where *h* is height above the ground measured in eye-heights. Andersen et al. (1999) measured participants' estimates of whether a collision would occur when watching simulations of decelerating self-motion toward a stationary lead object. They found that participants' were more likely to respond that a collision would have occurred for trials that had higher texture densities and therefore greater edge rates. Similarly, Rock and Harris (2006) found that both the presence of a ground plane and denser ground-plane textures resulted in shorter braking reaction times, more accurate estimation of initial braking forces, and fewer crashes with the target. Fajen (2005) compared the effects of edge rate and GOFR on participants' ability to stop at a stationary lead object and found that GOFR had a larger influence on participants' braking performance than edge rate.

The control model resulting from incorporating Equation 2 is also dependent on participants having knowledge about the size of the lead object. It is therefore possible that the selection of time headway is influenced by the actual size of the lead object such that, if actual size is not known (or specified), smaller objects could be followed at closer headways than larger objects. Indeed, both time-to-collision judgements and active braking for approaching objects of ambiguous sizes have been shown to be underestimated for smaller objects relative to larger ones (e.g., DeLucia et al., 1991, 2008; Andersen et al., 1999; Smith et al., 2001; Hosking and Crassini, 2011). Moreover, López-Moliner et al. (2007) have shown that the initiation of interceptive actions is critically dependent on prior knowledge regarding the size of the approaching object. Hosking and Crassini found that prior knowledge is important when judging the actual time-to-collision of a single approaching objects (Hosking and Crassini, 2010), but not when the judging relative TTC of two objects (Hosking and Crassini, 2011). We were interested to see if a similar effect of size occurred for headway selection such that smaller lead objects are followed more closely than larger lead objects.

In summary, research has shown that θ and $\dot{\theta }$ are important variables for timing interceptions, collision avoidance, braking, and the regulation of headway. However, previous investigations of headway maintenance have required participants to maintain a fixed following distance and have assumed that participants attempt to maintain the position of the lead object such that it subtends a fixed visual angle. As discussed above, other visual cues may be important for headway maintenance, particularly when participants are not required to hold a constant θ, but rather are allowed to vary their following distance over time. Furthermore, none of the previous research on headway maintenance has investigated the influence of ground texture, and given the importance of GOFR for judging speed and braking to avoid collision, it seems likely that ground texture might also influence headway maintenance.

In the following experiment, we asked participants to use a joystick to follow a computer simulated lead object. On each trial, the size of the lead object, its average velocity, and the presence of ground texture were manipulated. Participants were allowed to follow the lead object at any distance within a broadly defined minimum and maximum. It was hypothesized that (1) smaller objects will be followed at shorter following distances than larger objects, and (2) longer following distances will occur in the faster following speed conditions, particularly when ground speed information is saliently specified by the presence of a textured ground plane.

In addition to determining how the mean and standard deviation of following distances might vary as a function of object size, ground texture, and speed, we also modeled the joystick-controlled acceleration and velocity responses throughout each trial. Time-series analyzes were carried out in order to determine the control variables necessary for continuous adjustment of headway. In essence, we were comparing two alternate explanations for how humans maintain headway relative to lead objects, each derived from the simple relationship between following distance (*D*), following speed (*V*) and time headway (*T*); that is:

(4) D=T·V

In one model, it is assumed that *D* is kept constant (by maintaining a constant θ) and that *T* is allowed to vary (Equation 1). We compared this with an alternative model that assumes that people attempt to hold a constant *T*, and that *D* (i.e., the θ of the lead object) is free to vary as a function of *V* (see Equation 2). If *T* is an important determinant of user control when following lead objects, then Equation 2 shows that it would be necessary for participants to have access to information about own-speed. Therefore, Equation 2 should provide a better fit to the data when ground texture (and therefore GOFR) is available in the displays.

## 2. Materials and methods

### 2.1. Participants

Participants were 12 volunteers recruited from a NASA Ames participant database and students from San Jose University. There were a total of seven females and five males with a mean age of 27.3 years (*SD* = 8.8). Participants were required to have held a drivers licence for a minimum of two years. All had normal or corrected-to-normal visual acuity.

### 2.2. Apparatus and stimuli

Data collection took place in a quiet, dimly lit laboratory at the Human Systems Integration Division, NASA Ames. The main source of ambient light came from the computer monitor. Participants viewed computer generated displays with their head positioned in a forehead and chin rest located 83.4 cm from the computer monitor. At this viewing distance, the monitor subtended a visual angle of 29.5° vertically and 52.2° horizontally. During data collection, participants viewed the displays monocularly with an eye patch covering their non-dominant eye. Displays were generated, display presentation was controlled, and participants' responses were collected, using a Colfax FX700 workstation with a NVIDIA Quadro 4000 graphics card. The workstation drove a 60 cm Acer monitor with a resolution of 1980 pixels (vertically) by 1080 (horizontally), and a refresh rate of 120 Hz.

**Figure 1:**
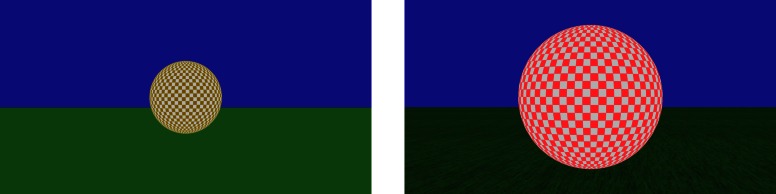
**Screen shots taken from the first frame in the displays at the beginning of a trial.** The left hand panel shows the smaller-sized (green-and-white checkered) lead object with no ground texture; the right hand panel shows the larger-sized (red-and-white checkered) lead object with ground texture.

Displays consisted of a green-colored ground and a blue-colored sky, with a spherical object that traveled away from the participants' viewpoint at a fixed elevation and azimuth that corresponded to the center of the computer monitor screen. The smaller object had a diameter of 120 cm and was textured with a green-and-white checker pattern; the larger object had a diameter of 240 cm and was textured with a red-and-white checker pattern (see Figure 1). The objects were displayed in two different ground texture conditions. In the no-texture condition, the ground plane appeared as a uniform green with no surface texture. In the texture condition, a green textured image was mapped onto the ground plane.

The speed at which the lead object traveled was determined by the sum of a constant, undisturbed speed and a perturbation speed, as defined below. In both texture conditions, a single (smaller or larger) object appeared to travel away from the participants' viewing point in one of two speed conditions. In the faster speed condition, the undisturbed speed of the lead object was 18 eye-heights/s (28.8 m/s^−1^); in the slower speed condition the undisturbed speed of the lead object was 9 eye-heights/s (14.4 m/s^−1^). An eye-height was defined as 1.6 m above the ground.

The speed of the lead object was perturbed by the sum of 12 harmonically independent sinusoids. The input perturbation (*I*) to the speed of the lead object was defined as:

$$I(t)=D\sum_{i=\;1}^{12}a_{i}\omega_{i}\cos(\omega_{i}t+\rho_{i}),$$

where $\omega_{i}=\frac{2\pi k_{i}}{60}$ rad/s. Table 1 shows the 12 values of *a* and *k* used to calculate ω. *D* was set to 0.7 m and the phase offset of each sine component (ρ_i_) was randomly varied from −π to π.

**Table 1:** **Magnitudes and frequencies used for the input perturbations to the speed of the lead object**.

***i***	***a*_*i*_**	***k*_*i*_**	**ω_*i*_ rad/s**
1	1.0	5	0.52
2	1.0	9	0.94
3	1.0	13	1.36
4	1.0	19	1.99
5	1.0	27	2.83
6	1.0	41	4.29
7	0.1	53	5.55
8	0.1	73	7.64
9	0.1	103	10.79
10	0.1	115	12.04
11	0.1	139	14.56
12	0.1	157	16.44

**Figure 2:**
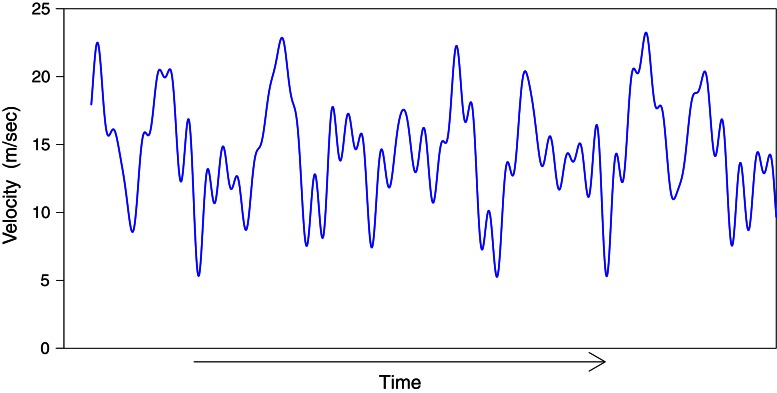
**Example of the perturbed speed of the lead-object as a function of time**.

The perturbation on the speed of the lead object made it appear that it was randomly accelerating and decelerating throughout the trial (see Figure 2). Participants used a joystick (B&G systems, F3) to follow the lead object by controlling either (1) the speed with which their view point (camera) translated in the median plane (i.e., velocity-control), or (2) the acceleration of their viewpoint (i.e., acceleration-control). The position of the joystick was sampled at 120 Hz.

### 2.3. Procedure and experimental design

Each trial began with the lead object stationary at a distance of six eye-heights (9.6 m) from participants' view point. When the participant initiated a trial by pulling the trigger on the joystick, the lead object began translating away from the view point. The magnitude of the velocity of the lead object was ramped up over the first 10 s of a trial by linearly scaling up the two speed components (i.e., speed condition and input perturbation) of that trial. Each trial lasted a total of 70 s. Participants were instructed to follow the lead object at what they determined to be a safe headway, with the constraints that they were not permitted to be so close to the lead object that its screen size was larger than the size of the monitor (i.e., a collision), nor so far away that headway exceeded 25 eye-heights (i.e., 40 m, whereupon another object would cut across the participant's path and occlude the participant's view of the lead-object). If a trial was terminated due to constraint violation, then it was repeated later in the experiment. Such constraint violations were only found to occur in the practice/familiarisation sessions, which are described below.

The experiment used a 2 (object size) × 2 (lead object speed) × 2 (ground texture) × 2 (control dynamics) within-subjects factorial design. There was one familiarisation/practice session and one experimental session for each of the control conditions (velocity and acceleration). The familiarization and practice sessions were used to ensure that participants were familiar with the task and the joystick dynamics. Participants in the familiarity session were encouraged to explore various headways and calibrate themselves to those headways that were either too close or too far from the lead object. Participants were then required to complete each trial of the practice session within the constraints of the task (see above) in order to proceed to the next trial. There was a 30 s break between trials. At the end of the practice session, participants had a short break and then completed the experimental session. The practice and experimental sessions were then repeated for the other control condition. Each participant was randomly assigned to complete either the velocity-control condition first followed by acceleration-control, or vice versa. All together the experiment took approximately 2.0 h to complete.

### 2.4. Data analyses

Time series of the speed perturbation to the lead object, joystick control output, and distance headway, were recorded at 120 Hz for each trial and for each participant. We analysed the data beginning 10 s after the start of the trial to ensure that any initial transient responses were skipped, and to exclude the initial period of lead-object acceleration. Two performance metrics were calculated from the remaining time-series data: (1) mean distance headway; and (2) the standard deviation (root mean squared) of distance headway. In order to examine participants' continuous control responses, we performed time-series analyses to determine how well the participants' accelerations could be modeled over time. The time series analyses were initially carried out by fitting the two alternate models to the data; that is:

1. An adaptation of Andersen and Sauer's (2007) Equation 1 where the θ′ of the lead object was assumed to be based on a constant θ, which we defined as the mean θ recorded over each trial (hereafter referred to as the θ_*D*_μ__ model); and
2. An adaptation of Andersen and Sauer's model that included Equation 2 to take into account the possibility that the θ′ of the lead object was assumed to be based on a constant time headway, which we defined as the average time headway recorded over each trial (hereafter referred to as the θ_*T*_μ__ model).

## 3. Results

### 3.1. Performance metrics

Separate three-way repeated-measures ANOVAs were performed on the velocity (1st order) and acceleration (2nd order) control data for each of the performance metrics described above.

#### 3.1.1. Analyses of velocity-control data

**Figure 3:**
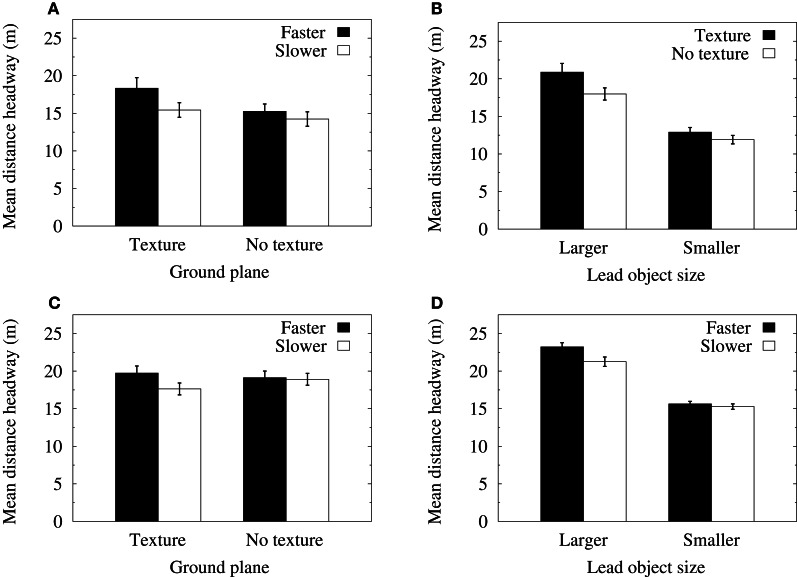
**(A)** Mean distance headways in the velocity-control condition as a function of the speed of the lead object and the presence of ground texture. **(B)** Mean distance headways in the velocity-control condition as a function of the size of the lead object and the presence of ground texture. **(C)** Mean distance headways in the acceleration-control condition as a function of the speed of the lead object and the presence of ground texture. **(D)** Mean distance headways in the acceleration-control condition as a function of the size of the lead object and lead object speed. Error bars indicate one standard error of the mean.

A repeated-measures ANOVA of mean distance headways in the velocity-control conditions revealed significant main effects for object size [*F*_(1, 11)_ = 70.71, *p* < 0.0001>] and lead object average speed [*F*_(1, 11)_ = 15.27, *p* < 0.01]. There were also significant two-way interactions between object size and ground texture [*F*_(1, 11)_ = 5.99, *p* < 0.05] and between ground texture and lead object average speed [*F*_(1, 11)_ = 5.31, *p* < 0.05]. Figure 3A shows that when ground texture was not present in the displays, mean distance headways were not significantly different for faster or slower lead object speeds. However, when the ground plane was textured, distance headways were significantly longer when the lead object was translating at a faster mean speed. As can be seen in Figure 3B, the larger lead objects were followed at longer distance headways than the smaller objects. Furthermore, there was a significant increase in distance headways for the larger lead object when ground texture was present in the displays. This latter effect did not occur when following the smaller object.

**Figure 4:**
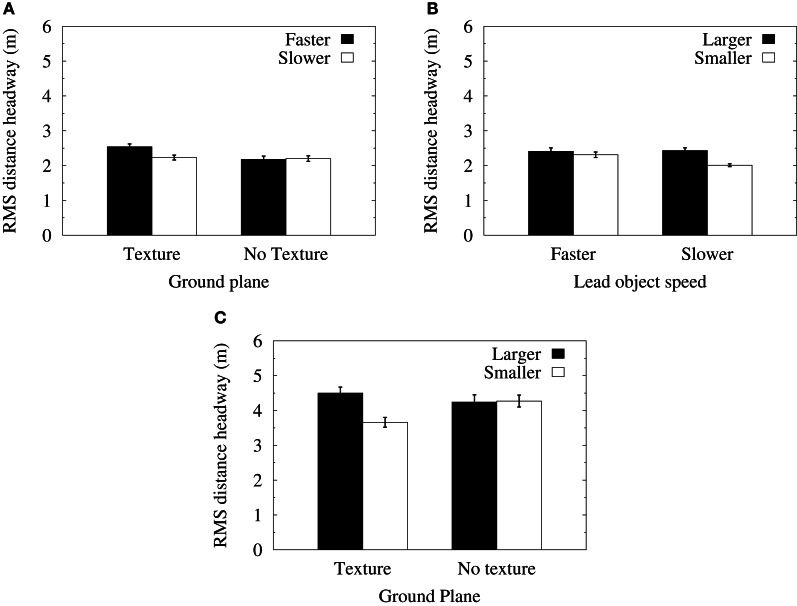
**(A)** Mean RMS of distance headways in the velocity-control condition as a function of the presence of ground texture and the speed of the lead object. **(B)** Mean RMS of distance headways in the velocity-control condition as a function of the size of the lead object and its speed. **(C)** Mean RMS of distance headways in the acceleration-control condition as a function of the size of the lead object and the presence of ground texture. Error bars show one standard error of the mean.

Root mean squared (RMS) data were calculated from the deviation of each distance headway data point (sampled at 120 Hz) from the mean distance headway in each of the experimental conditions. An ANOVA of RMS headway in the velocity-control conditions found significant main effects for object size [*F*_(1, 11)_ = 21.33, *p* < 0.001] and lead object average speed [*F*_(1, 11)_ = 5.21, *p* < 0.05]. The interactions between object size and the average speed of the lead object [*F*_(1, 11)_ = 8.9, *p* < 0.05] and between the lead object average speed and the presence of ground texture [*F*_(1, 11)_ = 5.23, *p* < 0.05] were also significant. Figure 4A shows that when a textured ground plane was present in the displays, RMS distance headways were significantly increased when the lead object had a faster average speed relative to when the lead object was translating at slower mean speeds. Figure 4B shows that RMS distance headways when following smaller objects were reduced in the slower speed conditions. Furthermore, a significant reduction in RMS distance headways for the smaller object relative to the larger object only occurred when the lead object was translating at slower mean speeds.

#### 3.1.2. Analyses of acceleration-control data

A repeated-measures ANOVA of mean distance headways in the acceleration-control conditions revealed significant main effects for object size [*F*_(1, 11)_ = 72.64, *p* < 0.0001] and lead object average speed [*F*_(1, 11)_ = 9.41, *p* < 0.01]. There were also significant two-way interactions between the size of the lead object and its average speed [*F*_(1, 11)_ = 6.02, *p* < 0.05] and between ground texture and lead object average speed [*F*_(1, 11)_ = 16.34, *p* < 0.01]. Consistent with the analyses of the velocity-control data, Figure 3C shows that when the ground plane was textured, mean distance headways were significantly longer when the lead object was traveling at faster speeds. Furthermore, Figure 3D shows that the larger lead objects were followed at longer distance headways than the smaller objects, and that there was a significant increase in distance headways when following the larger lead object at faster speeds.

An ANOVA of RMS distance headways in the acceleration-control conditions found significant main effects for object size [*F*_(1, 11)_ = 8.25, *p* < 0.01] and lead object average speed [*F*_(1, 11)_ = 6.59, *p* < 0.05]. There was also a significant interaction between object size and ground texture [*F*_(1, 11)_ = 6.51, *p* < 0.05]. Figure 4C shows that mean RMS distance headways were significantly reduced for smaller objects relative to larger objects in the textured ground plane condition. Furthermore, RMS distance headways for smaller objects decreased when ground texture was present, whereas RMS distance headways for larger objects did not vary significantly.

### 3.2. Time series modeling

Initial modeling was used to fit least squares regressions to the time-series data for each trial and for each participant. The regressions were used to determine the coefficients for each predictor variable of each of the θ_*D*_μ__ and θ_*T*_μ__ models described in Equations 1, 2 respectively, and also to determine the overall proportion of variance explained by each model. Regressions were carried out for both grouped data (i.e., data averaged over the 12 participants) and for each individual. For all analyses, the errors in each model were checked for normality and found to be Gaussian. In order to account for lag in user responses and the potential for different lags for each of the predictor variables, we first calculated the optimal lag between the predictor variables and the joystick response for each trial. Using cross-correlations between the predictor variables and the response, lags between 0 and 3 s were tested and the optimum lag for each predictor variable was then used in the model fitting.

To measure the predictive value of each model in describing participants' joystick control data, the variance of the statistically independent standardized prediction and error components of the model were calculated. The proportion of variance explained by each model (*R*^2^) for the group data are shown in Table 2. In order to compare models, we calculated Bayes Information Criterion (BIC) Scores (see Table 3) and Bayes factors (B) (see Table 4) for each model. BIC scores were calculated using reduced degrees of freedom to account for dependent samples (see Davey et al., 2013), resulting in more conservative estimates. The Bayes factor provided two measures of model performance: First, it provides a measure of which model is the best predictor of the data. Second, it provides a measure of the strength of evidence for a model relative to other models (see Lewandowsky and Farrell, 2011); a Bayes factor in the range of 3–10 provides moderate support for a model, whereas a Bayes factor greater than 10 indicates strong evidence for that model.

**Table 2:** **Model fits (*R*^2^) for the θ_*D*_μ__, θ_*T*_μ__, θ_*D*_*MA*__, and θ_*T*_*MA*__ models for both the acceleration-control and velocity-control conditions as a function of the size of the lead object, its speed, and the presence of ground texture**.

**Joystick control**	**Model**	**Small**				**Large**			
		**Slow**		**Fast**		**Slow**		**Fast**	
		**No texture**	**Texture**	**No texture**	**Texture**	**No texture**	**Texture**	**No texture**	**Texture**
Acceleration	$j\left(\frac{1}{\theta }-\frac{1}{\theta_{D_{\mu }}}\right)+k\dot{\theta }$	0.70	0.75	0.72	0.71	0.66	0.63	0.64	0.69
	$s\left(\frac{1}{\theta }-\frac{1}{\theta_{T_{\mu }}}\right)+t\dot{\theta }$	0.66	0.66	0.66	0.64	0.66	0.60	0.58	0.65
	$m\left(\theta -\theta_{D_{MA}}\right)+n\dot{\theta }$	0.88	0.87	0.88	0.88	0.89	0.86	0.81	0.89
	$f\left(\theta -\theta_{T_{MA}}\right)+g\dot{\theta }$	0.77	0.83	0.79	0.82	0.78	0.79	0.72	0.80
	$m\left(\theta -\theta_{D_{MA}}\right)+n\dot{\theta }+pV$	0.89	0.88	0.89	0.89	0.90	0.87	0.82	0.90
	$f\left(\theta -\theta_{T_{MA}}\right)+g\dot{\theta }+qV$	0.83	0.85	0.83	0.86	0.83	0.82	0.79	0.85
Velocity	$j\left(\frac{1}{\theta }-\frac{1}{\theta_{D_{\mu }}}\right)+k\dot{\theta }$	0.58	0.58	0.56	0.49	0.60	0.51	0.49	0.46
	$s\left(\frac{1}{\theta }-\frac{1}{\theta_{T_{\mu }}}\right)+t\dot{\theta }$	0.50	0.50	0.55	0.49	0.51	0.42	0.44	0.45
	$m\left(\theta -\theta_{D_{MA}}\right)+n\dot{\theta }$	0.62	0.62	0.65	0.56	0.60	0.51	0.53	0.49
	$f\left(\theta -\theta_{T_{MA}}\right)+g\dot{\theta }$	0.52	0.52	0.55	0.49	0.57	0.48	0.45	0.45

Also included are the R^2^ values obtained when own-velocity (V) was added as an additional parameter to the moving average models for the acceleration-control conditions only (see text for details).

**Table 3:** **BIC values^1^ for the θ_*D*_μ__, θ_*T*_μ__, θ_*D*_*MA*__, and θ_*T*_*MA*__ models for both the acceleration-control and velocity-control conditions as a function of the size of the lead object, its speed, and the presence of ground texture**.

**Joystick control**	**Model**	**Small**				**Large**			
		**Slow**		**Fast**		**Slow**		**Fast**	
		**No texture**	**Texture**	**No texture**	**Texture**	**No texture**	**Texture**	**No texture**	**Texture**
Acceleration	$j\left(\frac{1}{\theta }-\frac{1}{\theta_{D_{\mu }}}\right)+k\dot{\theta }$	−0.5656	−0.6531	−0.5987	−0.5818	−0.5055	−0.4649	−0.4780	−0.5498
	$s\left(\frac{1}{\theta }-\frac{1}{\theta_{T_{\mu }}}\right)+t\dot{\theta }$	−0.5055	−0.5055	−0.5055	−0.4780	−0.5055	−0.4275	−0.4041	−0.4916
	$m\left(\theta -\theta_{D_{MA}}\right)+n\dot{\theta }$	−1.0054	−0.9670	−1.0054	−1.0054	−1.0471	−0.9314	−0.7848	−1.0471
	$f\left(\theta -\theta_{T_{MA}}\right)+g\dot{\theta }$	−0.6931	−0.8382	−0.7368	−0.8108	−0.7144	−0.7368	−0.5987	−0.7602
	$m\left(\theta -\theta_{D_{MA}}\right)+n\dot{\theta }+pV$	−1.0410	−0.9992	−1.0410	−1.0410	−1.0867	−0.9608	−0.8046	−1.0867
	$f\left(\theta -\theta_{T_{MA}}\right)+g\dot{\theta }+qV$	−0.8320	−0.8921	−0.8320	−0.9252	−0.8348	−0.8046	−0.7306	−0.8921
Velocity	$j\left(\frac{1}{\theta }-\frac{1}{\theta_{D_{\mu }}}\right)+k\dot{\theta }$	−0.4041	−0.4041	−0.3817	−0.3109	−0.4275	−0.3301	−0.3109	−0.2834
	$s\left(\frac{1}{\theta }-\frac{1}{\theta_{T_{\mu }}}\right)+t\dot{\theta }$	−0.3204	−0.3204	−0.3709	−0.3109	−0.3301	−0.2491	−0.2660	−0.2746
	$m\left(\theta -\theta_{D_{MA}}\right)+n\dot{\theta }$	−0.4521	−0.4521	−0.4916	−0.3817	−0.4275	−0.3301	−0.3501	−0.3109
	$f\left(\theta -\theta_{T_{MA}}\right)+g\dot{\theta }$	−0.3400	−0.3400	−0.3709	−0.3109	−0.3928	−0.3015	−0.2746	−0.2746

1 Note: BIC values are 1.0e + 03.

Also included are the BIC values obtained when own-velocity (V) was added as an additional parameter to the moving average models for the acceleration-control conditions only (see text for details).

**Table 4:** **Bayes Factor scores (*B*) for the θ_*D*_μ__, θ_*T*_μ__, θ_*D*_*MA*__, and θ_*T*_*MA*__ models for both the acceleration-control and velocity-control conditions as a function of the size of the lead object, its speed, and the presence of ground texture**.

**Joystick control**	**Model**	**Small**				**Large**			
	**Slow**		**Fast**		**Slow**		**Fast**	
		**No texture**	**Texture**	**No texture**	**Texture**	**No texture**	**Texture**	**No texture**	**Texture**
Acceleration	$j\left(\frac{1}{\theta }-\frac{1}{\theta_{D_{\mu }}}\right)+k\dot{\theta }$	1.28	1.85	1.47	1.54	1.00	1.17	1.36	1.27
	$s\left(\frac{1}{\theta }-\frac{1}{\theta_{T_{\mu }}}\right)+t\dot{\theta }$	1.00	1.00	1.00	1.00	1.00	1.00	1.00	1.00
	$m\left(\theta -\theta_{D_{MA}}\right)+n\dot{\theta }$	8.03	6.84	8.03	9.00	9.55	8.16	4.89	10, 12
	$f\left(\theta -\theta_{T_{MA}}\right)+g\dot{\theta }$	2.19	4.00	2.62	4.00	2.39	3.63	2.25	3.06
	$m\left(\theta -\theta_{D_{MA}}\right)+n\dot{\theta }$	3.67	1.71	3.06	2.25	4.00	2.25	2.17	3.30
	$f\left(\theta -\theta_{T_{MA}}\right)+g\dot{\theta }$	1.00	1.00	1.00	1.00	1.00	1.00	1.00	1.00
	$m\left(\theta -\theta_{D_{MA}}\right)+n\dot{\theta }+pV$	1.15	1.14	1.16	1.16	1.18	1.13	1.09	1.18
	$f\left(\theta -\theta_{T_{MA}}\right)+g\dot{\theta }+qV$	1.78	1.25	1.49	1.61	1.65	1.33	1.73	1.73
Velocity	$j\left(\frac{1}{\theta }-\frac{1}{\theta_{D_{\mu }}}\right)+k\dot{\theta }$	1.19	1.19	1.02	1.00	1.23	1.18	1.10	1.02
	$s\left(\frac{1}{\theta }-\frac{1}{\theta_{T_{\mu }}}\right)+t\dot{\theta }$	1.00	1.00	1.00	1.00	1.00	1.00	1.00	1.00
	$m\left(\theta -\theta_{D_{MA}}\right)+n\dot{\theta }$	1.32	1.32	1.29	1.16	1.23	1.18	1.19	1.08
	$f\left(\theta -\theta_{T_{MA}}\right)+g\dot{\theta }$	1.04	1.04	1.00	1.00	1.14	1.12	1.02	1.00
	$m\left(\theta -\theta_{D_{MA}}\right)+n\dot{\theta }$	1.26	1.26	1.29	1.16	1.08	1.06	1.17	1.07
	$f\left(\theta -\theta_{T_{MA}}\right)+g\dot{\theta }$	1.00	1.00	1.00	1.00	1.00	1.00	1.00	1.00

Note: Bayes factors have been produced by comparing each model against the model with the lowest BIC score (i.e., the worst performing model). Therefore, the model with a B = 1 is the model being compared against.

Also included are the B values obtained when own-velocity (V) was added as an additional parameter to the moving average models for the acceleration-control conditions only (see text for details).

**Figure 5:**
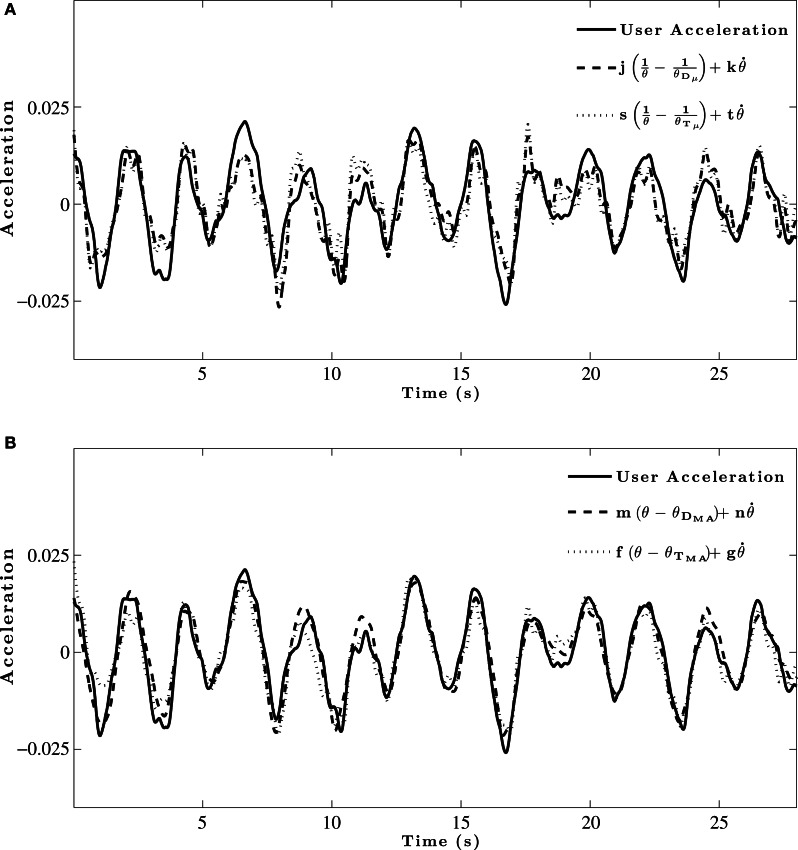
**Example model fit to group averaged acceleration data for a single trial. (A)** Initial model fitting comparing θ_*D*_μ__ and θ_*T*_μ__ models. **(B)** Model fits for comparison of optimized θ_*D*_*MA*__ and θ_*T*_*MA*__ models.

Sample fits of two of the models to group acceleration data are shown in Figure 5A. The results of the initial model fitting (see Table 2) found that the θ_*D*_μ__ model explained 63–75% of the variance in acceleration-control data, and 46-60% of the variance in the velocity-control data. For the θ_*T*_μ__ model, 58–66 % of the variance in the acceleration-control data and 42-55% of the variance in the velocity-control data was accounted for. However, while the *R*^2^ values (Table 2) and the BIC scores (see Table 3) both show an increase in the predictive power of the θ_*D*_μ__ model relative to the θ_*T*_μ__ model, the Bayes Factor Scores revealed that there was only weak evidence that the θ_*D*_μ__ model provided a better fit.

A potential limitation to the results thus far is that comparisons between models from which θ′ is derived (i.e., from either a constant *D* or a constant *T*) are problematic because of the differing statistical attributes of each derivation. That is, when θ′ is derived from a constant *T*, it includes all frequencies in the user control data. However, when θ′ is derived from a constant *D*, it contains no such frequencies at all. When participants are attempting to maintain headway, it seems likely that there would be some error in their ability to maintain θ′. Such error in their θ′ would result in θ′ having temporal dynamics within each trial that are independent of whether participants were relying on a constant *D* or constant *T*. Moreover, such variations in θ′ due to observer error may bias the model in favour of one that assumes that θ′ varies over time (i.e., one in which θ′ is based on a constant *T*). In order to control for this issue, we calculated θ′ from a moving average of either *D* or *T*. The moving average was essentially a low-pass filter which we calculated by determining an optimal window size such that θ′ contained the optimal frequencies when based on either a constant *D* or a constant *T*, thus ensuring that results of the time-series analyses were not biased towards a particular model. Using a brute force approach, the optimum window sizes were found to be 0.25 s for constant *T* and 3.5 s for constant *D*. Such window sizes provide some evidence that the participants were attempting to hold θ constant over a relatively long period of time and therefore indicate some support for them basing θ′ on a constant *D*. However, further analysis is warranted and we next compared the optimised models based on moving averages of either θ_*D*_μ__ or θ_*T*_μ__ (hereafter referred to as the θ_*D*_*MA*__ and θ_*T*_*MA*__ models, respectively) to the original θ_*D*_μ__ and θ_*T*_μ__ models.

Inspection of Table 2 shows both of the optimized models based on moving averages had larger *R*^2^ values than their original counter-part. The θ_*D*_*MA*__ model accounted for 81–89% of the variance in acceleration-control data; the θ_*T*_*MA*__ model accounted for 72–83% of the variance. Example fits of two of the models to group acceleration data are shown in Figure 5B. A comparison of all models in the acceleration-control condition, using the BIC scores and Bayes Factor scores shown in Tables 3 and 4 respectively, show that the θ_*D*_*MA*__ model was the best performing of the four, with Bayes Factor scores in many conditions approaching 10 (and in one case exceeding 10). Table 4 shows that the θ_*T*_*MA*__ model also performed significantly better than the base model, with moderate evidence for the strength of this model when a textured ground plane was present in the display. Table 4 also shows that when the θ_*D*_*MA*__ and θ_*T*_*MA*__ models are directly compared, the former provided a significantly better fit to the data when there was no texture available in the display. When texture was present, there was no significant difference between the two models. For the velocity-control conditions, Table 2 shows that 49–65% of the variance could be explained by the θ_*D*_*MA*__ model, and 45–57% the variance could be explained by the θ_*T*_*MA*__ model. Inspection of Tables 3 and 4 both show that calculating θ′ from the moving averages of either *D* or *TH* did not significantly improve their fit to the velocity data over and above that already shown for the original models. Bayes Factors derived from a direct comparison between the θ_*D*_*MA*__ and θ_*T*_*MA*__ models showed no significant difference in their fit to the velocity-control data.

In order to complete a final test of the role of GOFR, we compared the θ_*D*_*MA*__ and θ_*T*_*MA*__ models when the participants' own-speed (*V*) was included as an additional parameter in the model; that is,

(6) $$\ddot{x}=a(\theta -\theta^{\prime })+b\dot{\theta }+cV.$$

This comparison was made only for the acceleration-control data because the perfect correlation between *V* and velocity-control excluded it from further analysis. If perception of velocity is an important determinant of headway maintenance, it was predicted that the presence of ground texture, and hence GOFR, would improve this model as a predictor of following behavior. If this is the case, then the possible cost of including an additional degree of freedom in the model described in Equation 5 may be offset by its ability to better predict following behavior. The BIC method provides a weighted punishment for increasing model complexity and therefore has a greater preference for the simpler model. Because of this punishment term, the models with the extra parameter will be treated more conservatively in terms of their likelihood calculations. As would be expected, the inclusion of *V* as an additional parameter resulted in slightly better fits to the acceleration-control data (see the *R*^2^ values in Table 2), in particular for the θ_*T*_*MA*__ model. However, as can be seen in Table 4, there was little evidence that the addition of the *V* parameter significantly improved the fit of either the θ_*D*_*MA*__ or θ_*T*_*MA*__ models.

**Figure 6:**
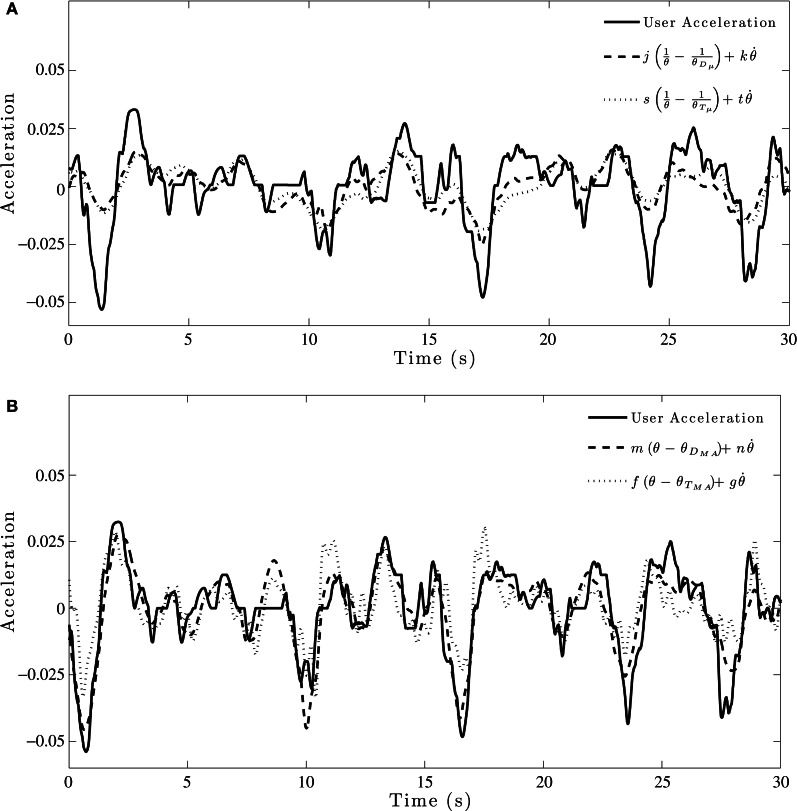
**Example model fit to individual acceleration-control data for a single trial. (A)** Initial model fitting comparing θ_*D*_μ__ and θ_*T*_μ__ models. **(B)** Model fits for comparison of optimized θ_*D*_*MA*__ and θ_*T*_*MA*__ models.

We also fit these models to individual data in both the acceleration-control and velocity-control conditions. A representative example of the model fitting in the acceleration-control conditions is shown in Figures 6A,B. As can be seen in Figure 6, individual response data were noisier than the group averaged data. However, while the individual data did not explain as much of the variance as that found for the group data, Table 5 shows that the pattern of results for individual data were similar to those found for the group data in both the velocity-control and acceleration-control conditions.

**Table 5:** **Proportion of variance explained (*R*^2^) by each model for individual response data in the velocity-control and acceleration-control conditions**.

**Joystick control**	**Model**	**s1**	**s2**	**s3**	**s4**	**s5**	**s6**	**s7**	**s8**	**s9**	**s10**	**s11**	**s12**	$\bar{x}$
Acceleration	$m\left(\theta -\theta_{D_{MA}}\right)+n\dot{\theta }$	0.72	0.66	0.64	0.60	0.59	0.74	0.80	0.66	0.70	0.70	0.80	0.67	0.70
	$f\left(\theta -\theta_{T_{MA}}\right)+g\dot{\theta }$	0.66	0.62	0.61	0.49	0.56	0.68	0.72	0.60	0.66	0.63	0.73	0.60	0.63
Velocity	$m\left(\theta -\theta_{D_{MA}}\right)+n\dot{\theta }$	0.33	0.37	0.32	0.34	0.30	0.36	0.26	0.30	0.36	0.37	0.45	0.34	0.34
	$f\left(\theta -\theta_{T_{MA}}\right)+g\dot{\theta }$	0.37	0.37	0.37	0.33	0.30	0.36	0.24	0.20	0.24	0.28	0.34	0.32	0.31

Note: s_n_ = participant number; $\bar{x}=R^{2}$ averaged across participants.

The time-series data were standardized to allow for comparisons of the obtained coefficients for each of the parameters in the θ_*D*_*MA*__ and θ_*T*_*MA*__ models. The mean standardized coefficients are shown in Table 6 collapsed across all conditions. As can be seen in Table 6, the weightings on control variables varied considerably as a function of the control dynamic of the task. For velocity-control, both the θ_*D*_*MA*__ and θ_*T*_*MA*__ models had larger weightings on $\dot{\theta }$ and smaller weightings on θ − θ′. For acceleration-control, the θ_*T*_*MA*__ model had an relatively even distribution of weightings on $\dot{\theta }$ and θ − θ′. In contrast, the θ_*D*_*MA*__ model had a larger weighting on θ − θ′, and a smaller weighting on $\dot{\theta }$.

**Table 6:** **Standardized coefficients for predictor variables of models for group performance as a function of ground texture**.

**Joystick control**	**Model**	**Parameters**	
		**θ − θ′**	$\dot{\theta }$
Acceleration	$m\left(\theta -\theta_{D_{MA}}\right)+n\dot{\theta }$	−0.77	−0.20
	$f\left(\theta -\theta_{T_{MA}}\right)+g\dot{\theta }$	−0.42	−0.57
Velocity	$m\left(\theta -\theta_{D_{MA}}\right)+n\dot{\theta }$	−0.22	−0.92
	$f\left(\theta -\theta_{T_{MA}}\right)+g\dot{\theta }$	0.16	−0.86

## 4. Discussion

Taken together, the results of the performance metrics analyses indicate that participants' choice of headway can be influenced by the size of the lead object, its speed, and the presence of ground texture. Given that GOFR influences passive braking judgements (Andersen et al., 1999) and active collision avoidance (Fajen, 2005; Rock and Harris, 2006), it is sensible that the presence of ground texture resulted in longer mean distance headways when traveling at faster speeds in our study. This effect of ground texture on mean following distances was significant in both the velocity-control and acceleration-control conditions. The finding that the presence of ground texture affected participants' choice of headway suggests that in addition to the contribution of θ and its derivatives, GOFR is an important source of information for headway maintenance. Ground texture also had an effect on the variance of participants' distance headways. In the velocity-control conditions, there was a significant difference in headway variance between faster and slower following speeds only when ground texture was present. Similarly, there was less variance in following distance for smaller lead objects than for larger objects in acceleration-control conditions when ground texture was present. The increase in following distance variance in the textured ground plane conditions suggests that participants were using GOFR as an additional source of information for controlling the relative distance between themselves and the lead object. GOFR would have allowed participants to distinguish whether the changing θ of a lead object was due to changing own-speed or changing lead-object speed. It is therefore possible that when GOFR is available participants are able to use a less conservative threshold for responding to changes in θ, and therefore ignore some changes in θ that presumably would not result in a collision.

With respect to the time-series modeling, the addition of information about own-speed via a textured ground plane in the acceleration-control conditions resulted in significant improvements to the predictive power of the θ_*T*_*MA*__ model. This latter finding was expected given that the time-headway model is dependent on *V*. For the better fitting θ_*D*_*MA*__ model, we found that the presence of ground texture did not result in any improvements to the amount of variance explained in either acceleration-control or velocity-control conditions, even when own-speed was included as an additional parameter. Overall, the negligible changes to the predictive power of the models on the basis of the availability of own-speed information demonstrates that participants' following behavior can be modeled more parsimoniously on the basis of visual angles alone or in combination with GOFR. Furthermore, when considering the combined results of the performance measures and the time-series modeling, it seems that if these models are reliably describing human perceptual-action systems then they capture nicely the proposal that the control variables used for controlling one's actions vary depending on the information available (e.g., see DeLucia et al., 2003; DeLucia, 2008).

**Figure 7:**
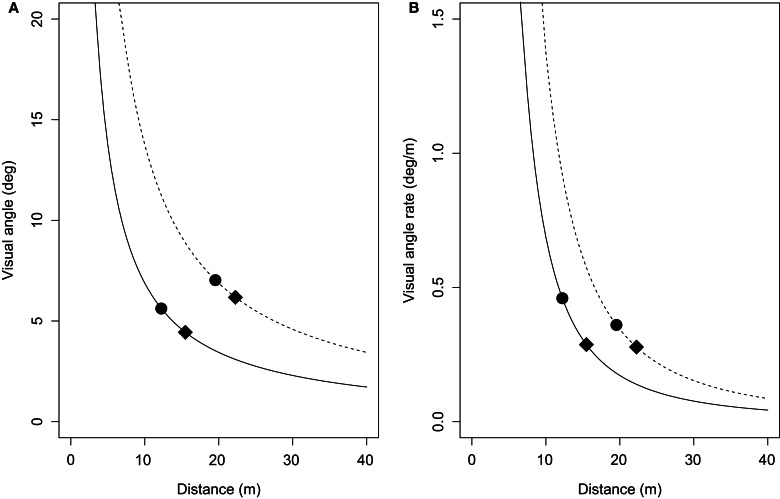
**(A)** Visual angle (in degrees) of the smaller (solid line) and larger (dashed line) lead objects as a function of their distance from the viewpoint. **(B)** Rate of change of visual angle (in degrees/m) of the same smaller and larger lead objects as a function of their distance from the viewpoint. Mean visual angles (and visual angle rates) of the smaller and larger objects are shown for the velocity-control condition (filled circles) and acceleration-control condition (filled diamonds). See text for details.

Consistent with previous reports of an effect of size when judging motion in depth (e.g., Andersen et al., 1999; Smith et al., 2001; DeLucia et al., 2008; Hosking and Crassini, 2011), a strong and consistent finding in this study was that participants followed smaller lead objects at a closer distance than larger lead objects. The results also found that following distances increased when travelling at faster speeds only for the larger objects and only when ground texture was present, further demonstrating that smaller lead objects may be responded to inappropriately. One possible explanation for these size-dependent biases can be derived from inspection of Figure 7. Figure 7A shows the average visual angles of the smaller and larger objects in both the velocity-control and acceleration-control conditions. These visual angles were calculated as a function of the average distance headway of the smaller and larger lead objects collapsed across all trials, and show that the mean θ of the larger object was 2–3° larger than that of the smaller object. However, inspection of Figure 7B shows that at the average distance headways found for the smaller and larger lead objects, the $\dot{\theta }$ of the lead objects were approximately equal in the acceleration control condition, and larger for the small object in the velocity-control conditions. This means that for an equivalent decrease in distance headway, relatively larger increases in the rates of change of $\dot{\theta }$ occurred for the smaller object. Given the importance of $\dot{\theta }$ for timed interceptions and collision avoidance (e.g., Smith et al., 2001; Hosking and Crassini, 2011), it seems plausible that increased salience of $\dot{\theta }$ for the smaller objects may have resulted in $\dot{\theta }$ begin given greater weighting than the GOFR in this case.

In line with the previous findings that acceleration-control is a more difficult task than velocity-control (McRuer and Jex, 1967), the magnitude of the variance in distance headway was larger for acceleration-control than for velocity-control. Furthermore, there were instances where some main effects and interactions noted in the velocity control conditions were not present in the acceleration-control conditions, and vice-versa. It is possible that these inconsistencies were due to the differences in the level of difficulty for the two types of control dynamics. Alternatively, it may be that participants use different sources of information, or apply different weightings to control variables, depending on the control dynamic. This latter explanation is supported in the time-series modelling, which showed that acceleration-control had relatively large weightings on θ′, whereas velocity-control seemed to put more weight on $\dot{\theta }$,.

An intriguing finding was the significant improvements to the model fits when an optimised moving-average was used. We propose that because there were no cues available in the displays (such as a reference size icon in a heads-up style display) providing a reference for θ′, participants' desired θ is subject to fluctuations over time. This is in contrast to previous models that have assumed that participants use a consistent desired θ, or a varying desired θ based on a constant-time headway. Our proposal is supported by the results which suggest that variations in participants' following distance are not due to them attempting to maintain a constant time-headway, but rather, are the result of their desired θ continuously changing, or drifting, from its original state according to what has been the average θ over a short period of time. It would be premature, however, to discount time-headway models of following behavior on the basis of the current results. This is because our displays did not provide any information that could have allowed participants to determine the actual sizes of the objects. Given that knowledge of object size is a variable of importance when determining headway (from Equation 2), the use of ambiguously sized objects in our experiments was likely to reduce the likelihood that the θ_*T*_ model would provide the best prediction of following behavior. It would be worth conducting a similar experiment in which participants were allowed to learn the actual sizes of the lead objects via either passive or active exploration of objects with the same sizes as those being simulated.

Perhaps most importantly, we were able to report good fits to participants' acceleration-control data, and reasonable fits to the velocity-control data even though participants were free to chose any headway at any time during the trial. These results are important because the time-series modeling suggests that when operators' headway is allowed to vary, they are best described as attempting to match the magnitude of θ to value that is not constant, but rather, varies over time. We also showed that participants tended to vary their mean headway depending on the speed that they were traveling. Taken together, these results demonstrate that while information about own-speed is used by controllers to set the desired headway to a lead object, the continuous regulation of headway is primarily influenced by the visual angle of the lead object and/or its rate of change, depending on the control dynamics of the system. Such a finding is consistent with previous reports that flexible strategies are used for selection of θ and/or $\dot{\theta }$ as information for intercepting objects (Lopez-Moliner and Keil, 2012), and reflects an optimal control criterion (Jagacinski and Flach, 2003) such that differential weightings are applied to different sources of information depending on the plant dynamics. In conclusion, our ability to navigate through a three dimensional world is a complex task relying on multiple sources of information. As in other judgements of motion in depth, the information used for controlling headway to other objects in the environment seems to be dependent on the constraints of the task and different strategies of control.

### Conflict of interest statement

The authors declare that the research was conducted in the absence of any commercial or financial relationships that could be construed as a potential conflict of interest.
